# Rosette Cardiac MR Fingerprinting for Simultaneous T_1_ , T_2_ , $T_{2}^{*}$, and Fat Fraction Mapping Using a Multi‐Echo Deep Image Prior Reconstruction

**DOI:** 10.1002/mrm.70299

**Published:** 2026-02-09

**Authors:** Evan Cummings, Gastao Cruz, Jacob Richardson, Sydney Kaplan, Jesse Hamilton, Nicole Seiberlich

**Affiliations:** ^1^ Radiology University of Michigan Ann Arbor Michigan USA; ^2^ Biomedical Engineering University of Michigan Ann Arbor Michigan USA

**Keywords:** cardiac MRI, deep learning reconstruction, MR fingerprinting, quantitative MR

## Abstract

**Purpose:**

Quantitative mapping of cardiac tissue properties is used clinically in diagnosis and monitoring of a wide variety of cardiac pathologies. Cardiac Magnetic Resonance Fingerprinting (cMRF) enables rapid and simultaneous quantification of multiple parameters in the myocardium from a single scan. In this work, a multi‐echo cMRF acquisition is combined with a deep image prior framework to reconstruct cardiac T_1_, T_2_, $T_{2}^{*}$, and fat fraction maps.

**Methods:**

A 2D, single‐breathhold, ECG‐gated rosette trajectory cMRF sequence was deployed to sensitize the signal to T_1_, T_2_, $T_{2}^{*}$, and fat off‐resonance effects. Data were processed using a deep image prior reconstruction trained with the cMRF encoding model to generate images consistent with the acquired k‐space data. These images were used in curve fitting and pattern matching algorithms to generate T_1_, T_2_, $T_{2}^{*}$ and fat fraction maps. The technique was validated using numerical simulations, standard phantoms, and 28 healthy subjects.

**Results:**

In phantoms, good agreement was observed between the proposed technique and gold‐standard reference measurements. In healthy subjects, measurements made with the deep image prior (DIP) reconstruction agreed with clinical cardiac measurements and demonstrated smaller voxel‐level variance in a healthy population compared to iterative low‐rank and direct matching reconstructions.

**Conclusion:**

The multi‐echo cMRF acquisition coupled with a DIP reconstruction enables the simultaneous quantification of T_1_, T_2_, $T_{2}^{*}$, and fat in the heart and demonstrates good agreement with conventional mapping approaches in phantom and in vivo experiments. Additionally, the DIP reconstruction provides accurate measurements with a lower voxel‐level variance compared with direct gridding and iterative low‐rank reconstruction methods.

## 1 Introduction

Quantitative cardiac tissue property mapping is used clinically in the diagnosis and monitoring of a wide variety of diseases. Multiple tissue properties can be assessed using MRI, including T_1_, T_2_, $T_{2}^{*}$, and fat fraction, and each may change in the presence of myocardial pathology [1, 2, 3, 4, 5]. In clinical practice, each of these tissue property maps is collected in a separate scan, requiring a separate breathhold, which results in long protocols and mis‐registered maps. Moreover, it is difficult to isolate the encoding for any one of these properties, as any measurement is impacted by multiple magnetic properties. For example, T_1_ measurements may be influenced by both T_2_ and proton density fat fraction (PDFF) [6, 7]; meanwhile, fat fraction measurements are impacted by T_1_ and $T_{2}^{*}$ [8, 9]. While methods for correcting these effects exist, they are often limited to correcting for a single factor and require additional maps to be collected and co‐registered. An ideal approach would acquire these maps simultaneously and reconstruct them using a comprehensive signal model, as this would allow for correction of confounding effects without registration or collection of additional data.

Magnetic Resonance Fingerprinting (MRF) has been proposed as an efficient method for multiparametric mapping [10, 11, 12]. Compared to traditional tissue property mapping, MRF uses a transient‐state acquisition to encode information from multiple tissue properties. A unique signal evolution can then be modeled for each T_1_ and T_2_ pair and matched to the acquired signal, enabling the collection of multiple maps from a single scan. By mapping multiple properties simultaneously, it can reduce the number of scans required for patients, potentially improving patient comfort and reducing the time required for cardiac protocols. Additionally, by using a signal model calculated via the Bloch equations, modeling errors can be reduced. For instance, when measuring T_1_ with MOLLI, it is assumed that magnetization recovers completely over eight heartbeats, which may not be the case in tissues with long T_1_ values, causing an underestimation of T_1_. In cMRF, the incomplete recovery is modeled during dictionary simulation, reducing its sensitivity to these errors and potentially enabling more accurate measurements [12].

The original implementation of MRF mapped only T_1_ and T_2_ [10], but recent approaches have investigated methods to acquire additional tissue property maps using this framework. A common approach is to integrate a multi‐echo acquisition into the MRF pulse sequence, such that fat fraction and $T_{2}^{*}$ information is embedded in the MRF signal. Liu et al. [13] and Jaubert et al. [14] have used rosette and multi‐echo radial cMRF sequences to simultaneously estimate T_1_, T_2_, and fat fraction in the heart. In the liver, Jaubert et al. [15] and Fujita et al. [16] have used multi‐echo radial MRF sequences to estimate T_1_, T_2_, $T_{2}^{*}$, and fat fraction. More recently, multi‐echo MRF sequences have been adapted for cardiac applications as well, utilizing motion correction to overcome scan time limitations [17].

One challenge with multi‐echo MRF is the need to balance the number of excitations available for T_1_ and T_2_ mapping and the length of the gradient echo readout for $T_{2}^{*}$ and fat fraction mapping. Compared to conventional cMRF for T_1_ and T_2_ mapping, multi‐echo cMRF requires longer readouts to capture $T_{2}^{*}$ decay in the myocardium. However, use of longer readouts requires a reduction in the number of excitations for encoding T_1_ and T_2_ properties, assuming that the diastolic acquisition window and breathhold duration are kept consistent to minimize motion. While this challenge can be solved by collecting data in a free‐running fashion and including motion correction in the reconstruction, this can lead to measurement errors in T_2_ and $T_{2}^{*}$ due to through‐plane motion [17], and 3D acquisitions require several minutes to fully capture both contrast and motion information in three spatial dimensions [18].

Recently, Hamilton et al. introduced a Deep Image Prior (DIP) reconstruction for cMRF data [19]. In this work, the DIP reconstruction was applied to cMRF data collected in a shortened acquisition of 5 heartbeats with a 150 ms diastolic acquisition window (comprised of only 140 data readouts, instead of the 750 which are typically collected in cMRF). Despite the reduction in cMRF excitations, the DIP reconstruction produced accurate and precise T_1_ and T_2_ maps. Given that the number of data readouts used in that work is similar to that which can be collected using a multi‐echo readout sufficient to encode myocardial $T_{2}^{*}$ values, the hypothesis of this work is that the combination of the DIP reconstruction and rosette cMRF data could enable accurate and precise mapping of T_1_, T_2_, $T_{2}^{*}$, and PDFF simultaneously.

This work introduces a single‐breathhold, 15 heartbeat rosette cMRF sequence for encoding T_1_, T_2_, $T_{2}^{*}$, and PDFF, and pairs it with a novel multi‐echo deep image prior reconstruction method. The performance of the multi‐echo cMRF sequence combined with DIP reconstruction is compared to conventional direct matching and iterative locally low‐rank reconstructions [20]. Multi‐echo cMRF data were acquired at 1.5 T to measure relaxation values in the ISMRM/NIST phantom and PDFF values in an in‐house oil/water data; these data were reconstructed with each method to assess accuracy and precision. Finally, the multi‐echo cMRF sequence with DIP reconstruction was deployed in 28 healthy subjects and compared to both existing cMRF reconstruction methods as well as conventional myocardial mapping techniques to assess the agreement between these approaches.

## 2 Methods

### 2.1 Design of Rosette Trajectory

**FIGURE 1:**
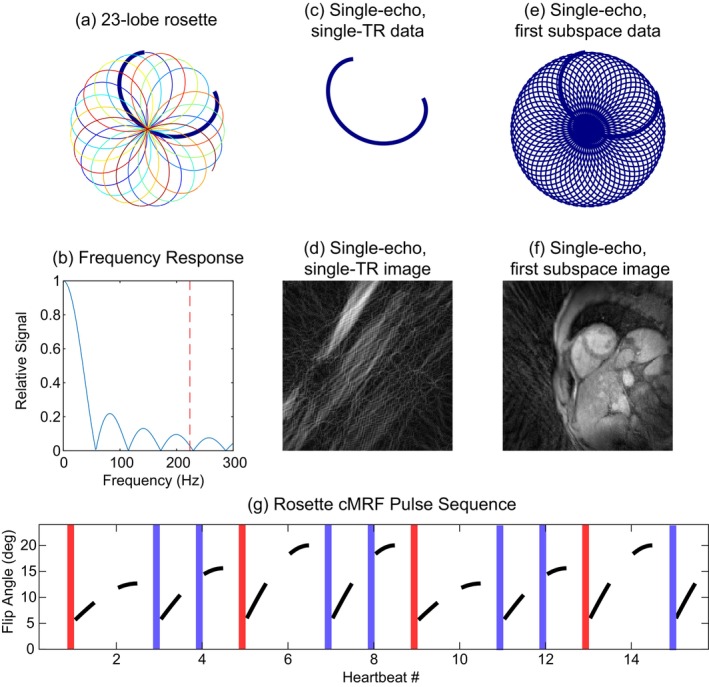
(a) The 23‐lobe rosette used for this study. (b) A plot of the response function of this rosette as a function of off‐resonance frequency, with the off‐resonance frequency of fat at 1.5 T shown as a dotted line at 220 Hz. The relative intensity at this frequency is 3% of the response for on‐resonance spins, corresponding to an attenuation of −31 dB. (c) One “echo,” or k‐space center crossing, from the rosette data. When reconstructing images, the rosette is split up into single echoes, and a separate image is generated from each echo via a gridding operation. (d) An image reconstructed from a gridding a single rosette echo. (e) A fully‐sampled k‐space for an echo, which can be created by combining data over multiple readouts of the cMRF signal. SVD compression [22] may be used to reduce variation between shots due to the transient‐state MRF sequence. (f) An image reconstructed from this data via direct gridding, which has residual aliasing artifacts. (g) A diagram of the rosette cMRF sequence used in this study, with a plot of the flip angles used; a red bar denotes heartbeats that were preceded by an inversion, and blue bars represent heartbeats that were preceded by a T_2_‐preparation pulse (alternating between T2‐prep TE = 30 and 80 ms).

The rosette trajectory [21] (shown in Figure 1) has several parameters which can be selected to improve its $T_{2}^{*}$ and fat fraction encoding power, including its total readout length, timing of the echoes (where the trajectory returns to the center of k‐space), the angular extent of each lobe, and the number of echoes. For this work, the rosette readout length was selected to be 18.4 ms based on SCMR guidelines [1] for cardiac $T_{2}^{*}$ mapping. The echo times for the rosette were selected to be close to N‐point Dixon echo times for optimal encoding of the fat signal [21, 23, 24]. The angular distance covered by each lobe was set to 117.4° to approximate the golden angle to maximize incoherence between subsequent echoes [25]. Finally, the number of lobes was maximized to best sample the $T_{2}^{*}$ decay curve; in this work, 23 lobes were sampled for a total of 24 echoes. Using the parametrization by Noll [21], *k*_max_ = 320 m^−1^, *ω*_1_ = 11.5, *ω*_2_ = 7.5. The gradient waveform was calculated using the time‐optimized method developed by Lustig et al. [26]. During gridding, the rosette readout is split using the method described by Bush et al. [27] and shown in Figure 1; this divides the rosette at the farthest extent of k‐space, creating images from each pass through the center of k‐space. The first and final echoes were excluded from reconstruction, as each is undersampled, covering only the initial pass out from or final pass back to the center of k‐space, rather than a full sweep from k‐space edge to edge.

### 2.2 DIP Pipeline for the Reconstruction of Tissue Property Maps

**FIGURE 2:**
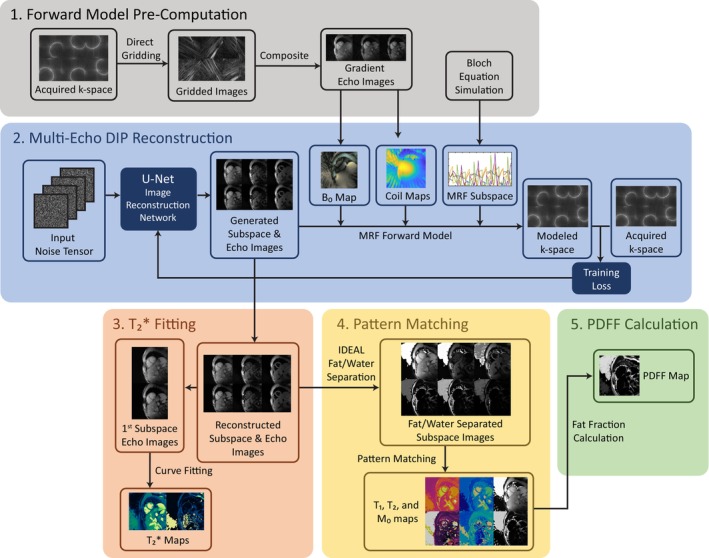
A diagram of the multi‐echo DIP reconstruction pipeline, consisting of five steps. (Part 1) The off‐resonance and coil maps required for later steps are pre‐calculated from a direct gridding reconstruction. (Part 2) Images for each echo of each cMRF subspace are reconstructed using the Deep Image Prior technique. (Part 3) $T_{2}^{*}$ decay curves are fit voxelwise along each echo to yield $T_{2}^{*}$ maps for both fat and water separately, using the dual‐$T_{2}^{*}$ method proposed by Chebrolu et al. [9] (Part 4) Fat/water separation is performed using IDEAL, and then pattern matching is used to create T_1_ and T_2_ maps from the fat/water separated subspace images. (Part 5) The *M*_0_ maps from pattern matching are then used to calculate the fat fraction map.

The proposed reconstruction consists of five main steps, as shown in Figure 2. First, the cMRF dictionary is generated for a fixed set of T_1_ and T_2_ values using subject‐specific ECG timings, and the off‐resonance and coil sensitivity maps are computed from the acquired cMRF data. Second, a convolutional neural network is trained to generate images for each cMRF subspace and echo time that are consistent with the acquired k‐space data [19]. Third, the echo images are used to estimate fat/water separated $T_{2}^{*}$ maps [9]. Fourth, iterative decomposition of water and fat with echo asymmetry and least squares estimation (IDEAL) [28, 29] is applied to the subspace‐echo images to generate separate fat/water images, and pattern matching is used to estimate fat/water separated T_1_ and T_2_ maps from these components. Finally, a fat fraction map is calculated from the matched spin density values. Each reconstruction step is described in more detail below.

### 2.3 Pre‐Computation of cMRF Encoding Operations

First, the elements of the cMRF encoding matrix are pre‐computed, including the density compensation function, the cMRF dictionary, the cMRF‐dictionary‐based compression matrix, off‐resonance map, and coil sensitivity maps. No additional scans are required; each of these matrices can be computed from the raw cMRF data or sequence parameters. The cMRF dictionary for the rosette acquisition is generated using a Bloch equation simulation and includes 28 010 combinations of entries for T_1_ = (10:10:2000, 2020:20:3000) and T_2_ = (6:2:100, 105:5:300, 310:10:500, 540:40:1200). The dictionary accounts for the subject's cardiac rhythm and corrects for inversion efficiency and slice profile effects [30]. The compression matrix is calculated using the first 10 singular vectors of the dictionary [22]. The density compensation function is calculated for each echo of the fully‐sampled rosette trajectory using the min‐max method described by Fessler et al. [31]. Next, the echoes associated with the first MRF subspace are reconstructed to compute coil sensitivity maps and an off‐resonance map. The echo images are reconstructed by applying SVD compression over sequence repetitions, and then dividing the rosette trajectory into echoes using the method proposed by Bush et al. [27], which yields a fully‐sampled k‐space for each echo. The off‐resonance map is computed from these echo images using the graph‐cut method [29]. This method for off‐resonance mapping is similar to the method proposed by Hu et al. [32], but divides the rosette trajectory at the maximum extent of k‐space rather than the center, and uses the graph‐cut algorithm for *B*_0_ mapping rather than the ADMM algorithm. Coil sensitivity maps are computed from these echo images after applying off‐resonance correction.

### 2.4 Deep Image Prior Image Reconstruction

**FIGURE 3:**
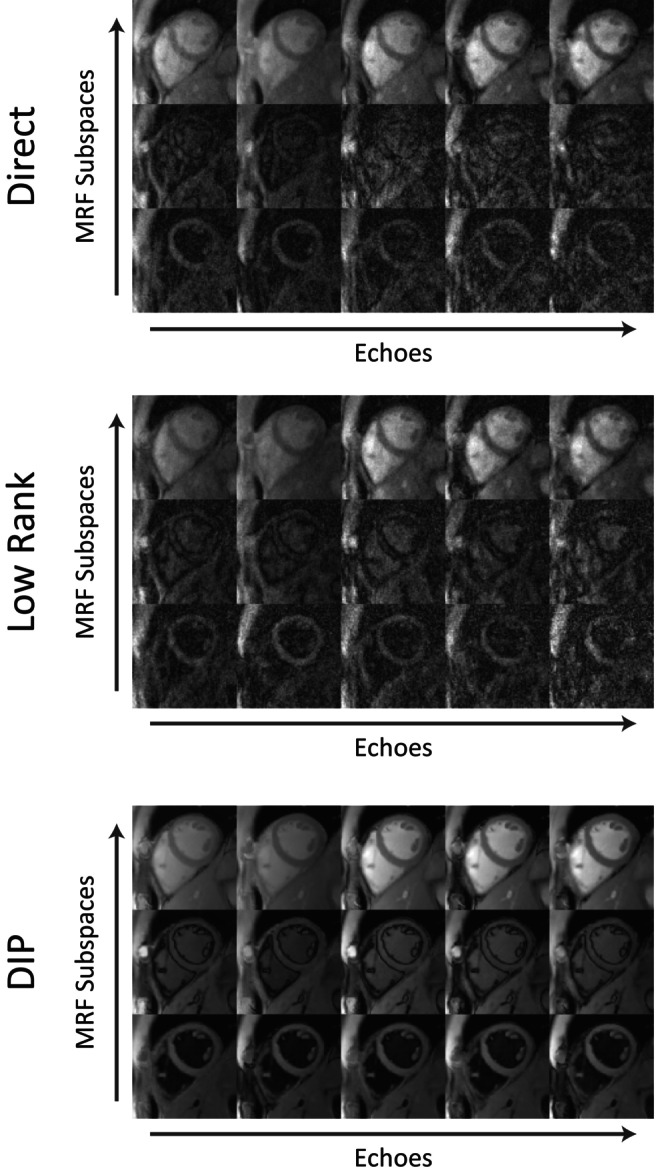
Subspace‐echo images from one volunteer reconstructed using the three methods examined in this work (direct gridding, top; iterative low‐rank method, middle; and DIP, bottom). In each set, the vertical axis corresponds to the subspace dimension, with each set containing a contrast associated with a cMRF subspace. The horizontal axis corresponds to the echo dimension, with each subspace having several echoes associated with it. Shown is a subset of the full subspace‐echo image set, with only 3/10 cMRF subspaces (compressed from 180 original readouts) and 5/22 echoes for each set. While there are additional processing steps between these images and the final tissue property maps, most of these operations are applied voxelwise; thus, the quality of this image set is strongly tied to the quality of the resulting maps.

Next, the DIP reconstruction process is initiated. The architecture of the DIP image reconstruction network is similar to that described by Hamilton et al. [19], and shown in Figure S1. The network used a dropout rate of 7%; dropout causes a fraction of neural network connections to be ignored at each iteration of training, which improves robustness to overfitting and acts as a regularization parameter for the DIP reconstruction. Two major modifications were made to the network to enable reconstruction of rosette cMRF data. In this work, the DIP network reconstructs “cMRF subspace‐echo images” (shown in Figure 3) along both the cMRF subspace (compressed TR) dimension and the echo dimension, such that information about the T_1_ and T_2_ contrast is contained in the subspace dimension and information concerning $T_{2}^{*}$ and fat/water content is contained in the echo dimension. Second, the DIP reconstruction was modified to include the off‐resonance map in the MRI encoding matrix. This correction is performed by multiplying the echo images by the expected phase progression prior to computation of the loss function, such that the network generates images without field inhomogeneity effects. The inclusion of an off‐resonance correction accelerates network training and mitigates susceptibility artifacts, as shown in Figure S2. Finally, only the image reconstruction network is used in this work; dictionary generation is performed with Bloch equation simulation, and parameter estimation is performed in the subsequent steps.

### 2.5 $T_{2}^{*}$ Mapping

Following the DIP reconstruction, a voxelwise dual‐exponential curve fitting algorithm is applied across the echo images from the first cMRF subspace to estimate fat/water separated $T_{2}^{*}$ values [9]. $T_{2}^{*}$ values are limited to lie between 2.2 and 200 ms.

### 2.6 T_1_ and T_2_ Mapping

To estimate T_1_ and T_2_ maps, IDEAL [28, 29] is first used to separate the fat and water components from the cMRF subspace‐echo images. The implementation is available in the ISMRM Fat‐Water Toolbox. This step removes $T_{2}^{*}$ decay or fat/water dephasing effects at the time of the first echo, yielding cMRF subspace images which are weighted only by T_1_ and T_2_. Following this step, pixel‐wise dot product pattern matching is performed on the IDEAL‐separated cMRF subspace images and the cMRF dictionary to estimate T_1_ and T_2_ maps for both the fat and water components.

### 2.7 PDFF Mapping

In addition to T_1_ and T_2_ maps, the pattern matching method estimates spin density maps for fat and water. These are used to compute the PDFF map using a noise‐robust method [8].

### 2.8 Data Collection

The cMRF pulse sequence used in this work is a variant of the FISP cMRF sequence proposed by Hamilton et al. [30], with flip angles increased to lie between 5.7° and 20.0° to compensate for the relaxation that occurs due to the increased TR. The rosette cMRF sequence used the following parameters: TR = 20.4 ms, TE_1_ = 1.74 ms, ΔTE = 0.79 ms, rosette echo train length = 22, FOV = 300 × 300 mm, voxel size = 1.56 × 1.56 mm^2^, 8 mm slice thickness, 180 excitations (12 per heartbeat, 245 ms acquisition window), flip angle range of 5.7°–20° in a sinusoidal pattern shown in Figure 1, pulse duration = 800 μs, TBP = 2.0, gradient system limits of 43 mT/m with a slew rate of 180 mT/m/s, and ADC sampling time = 2.5 μs. All data were acquired on a 1.5 T Sola Scanner (Siemens Healthineers, Erlangen, Germany). Data were acquired over 15 heartbeats during a breathhold with electrocardiograph (ECG) gating. Trajectory measurement was performed once prior to scanning [33]; the trajectory resulting from this measurement was used during the 16‐month period of data acquisition for this study. SAR limits were not a concern, as scanning delays between heartbeats allow for sufficient dissipation of energy.

### 2.9 Numerical Simulations

Numerical simulations on the MRXCAT phantom were used to assess the ability of the rosette cMRF sequence and DIP reconstruction to capture pathological T_1_, T_2_, $T_{2}^{*}$, and PDFF values. A simulation using values expected in healthy myocardial tissue, specifically T_1_ = 950 ms, T_2_ = 48 ms, $T_{2}^{*}$ = 30 ms, and PDFF = 1%, was performed. Four additional simulations were performed, with a pathological value (T_1_ = 1250 ms, T_2_ = 65 ms, $T_{2}^{*}$ = 15 ms, and PDFF = 10%) assigned to all of the pixels of the myocardium for each of the tissue properties of interest. An additional experiment, described in detail in Figure S3, was used to determine the influence of confounding effects on measurements made with each of the three reconstructions.

### 2.10 Phantoms

Phantom scans were performed on the ISMRM/NIST MRI system phantom and an in‐house peanut oil/water agar gel phantom [13]. Ground‐truth T_1_ values were measured using an inversion recovery sequence with inversion times (TI) = 24, 100, 200, 400, 800, 1600, and 3200 ms. Ground‐truth T_2_ values were measured using a single‐echo spin‐echo T_2_ sequence with TE = 10, 20, 40, 60, 100, 150, and 200 ms. Ground‐truth $T_{2}^{*}$ and PDFF values were measured using a gradient echo sequence with TE = 10.0, 13.94, 17.88, 21.82, 25.76, 29.70, 33.64, 37.58, 41.52, 45.46, 49.40, and 53.34. Fully‐sampled Cartesian data were reconstructed via Fourier transform, and maps were fit using an exponential model. Rosette cMRF data were acquired using a simulated ECG signal with an RR interval of 1000 ms. A linear regression was computed between the values measured using rosette cMRF and ground‐truth measurement for each property. T_1_, T_2_, and $T_{2}^{*}$ were assessed in the NIST phantom and PDFF in the oil/water phantom. Phantom spheres with T_2_ > 200 ms and $T_{2}^{*}$ > 80 ms were excluded from this analysis, as physiological values of interest fall below these limits.

### 2.11 Healthy Subjects

**FIGURE 4:**
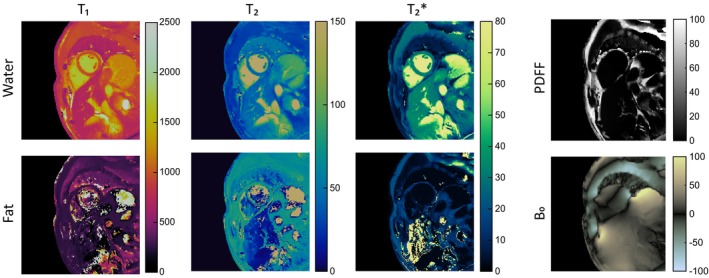
Full set of fat/water separated tissue property maps for T_1_, T_2_, $T_{2}^{*}$, and fat fraction collected in a representative healthy subject and reconstructed using the proposed DIP method.

In an IRB‐approved study, rosette cMRF data were collected in a mid‐ventricular short‐axis slice in 28 healthy subjects. A complete set of resulting maps from one volunteer is shown in Figure 4. In addition, the DIP reconstruction, images were also reconstructed using direct gridding of the undersampled images (as in the original implementation of MRF) and an iterative conjugate gradient reconstruction with locally low‐rank and spatial total variation regularization [20] to compare the proposed multi‐echo DIP framework with existing cMRF reconstruction approaches.

For each subject, conventional maps were also acquired using the Siemens Myomaps product sequences, specifically MOLLI for T_1_ mapping, T_2_‐prep bSSFP for T_2_ mapping, and a black‐blood gradient echo (GRE) for $T_{2}^{*}$ mapping; reference fat fraction maps were reconstructed offline from the GRE data using sensitivity encoding (SENSE) [34] and graph‐cut IDEAL [29]. Sequence parameters for conventional maps are reported in Table S1. A region of interest was selected over the entire mid‐ventricular myocardium in each subject. For each map, paired *t*‐tests with Holm‐Bonferroni corrections were performed to determine agreement between rosette cMRF maps reconstructed with each approach, and the conventional maps. Regional variations were assessed using the six mid‐ventricular American Heart Association (AHA) segments [35]; paired‐sample *t*‐tests with Bonferroni correction were used to identify segments that differed from the value measured over the whole myocardium. Bland–Altman analyses were used to determine agreement between maps generated using the DIP reconstruction and reference methods for T_1_, T_2_, $T_{2}^{*}$, and fat fraction measurements. In addition, a susceptibility artifact appears in the $T_{2}^{*}$ map in several subjects; if the inferior segment had a mean value lower than 20 ms, the subject was considered to have a localized artifact. The number of subjects exhibiting such an artifact was noted.

## 3 Results

### 3.1 Numerical Simulations

**FIGURE 5:**
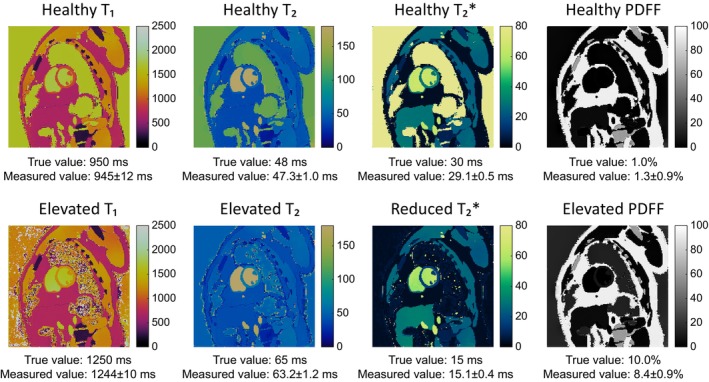
Results from the MRXCAT simulations. (top row) Results from the simulation of a healthy case. (bottom row) Results from the relevant map in each of the four pathological simulations.

Results from the MRXCAT experiment are shown in Figure 5, showing maps of the healthy case and the relevant tissue property map from each of the four pathology experiments. In T_1_, differences between true and measured values are less than 10 ms; in T_2_ and $T_{2}^{*}$, the differences are less than 2 ms. In PDFF, the healthy case shows agreement within 0.5%, while the pathological case slightly underestimates by 1.6%.

Results from the numerical phantoms are shown in Figure S3. Resulting magnetic property maps and PCC heatmaps are shown for each reconstruction. The PCC is used to determine the degree to which a simulated value affects a measured value. For on‐target effects (e.g., the effect of simulated T_1_ value on the measured T_1_ value), each property shows a statistically significant correlation; for off‐target effects (e.g., the effect of simulated T_1_ on measured PDFF), no statistically significant correlations are observed.

### 3.2 Phantoms

**FIGURE 6:**
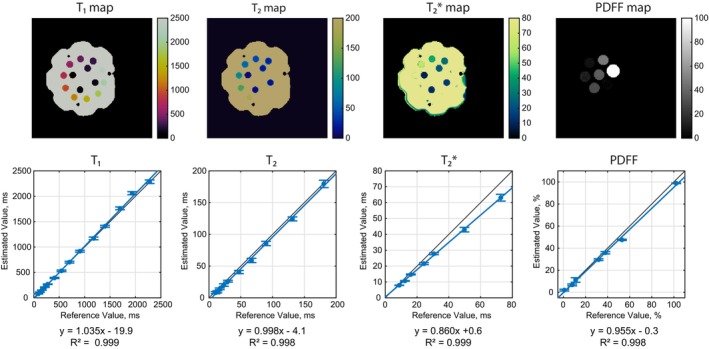
Regressions of measurements on the ISMRM/NIST MRI system phantom and the home‐built PDFF phantom.

Figure 6 shows a set of plots comparing the T_1_, T_2_, $T_{2}^{*}$, and PDFF values measured using the proposed rosette cMRF sequence on the ISMRM/NIST phantom and the home‐built fat/water phantom to ground truth values. There is excellent agreement between the experimental measurements and the ground truth reference measurements (DIP *R* ^2^ = 0.999 for T_1_, 0.998 for T_2_, 0.999 for $T_{2}^{*}$, and 0.998 for PDFF).

### 3.3 Healthy Subjects

**FIGURE 7:**
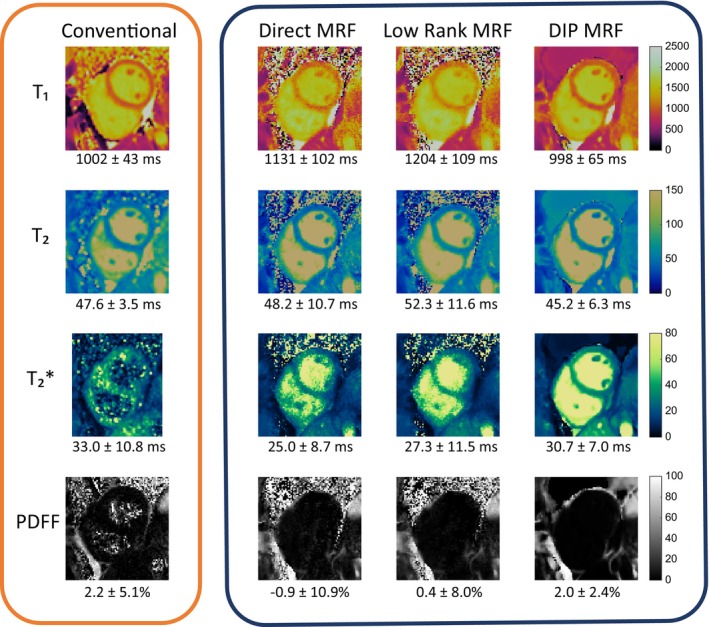
Conventional maps, direct reconstruction cMRF, iterative low‐rank reconstruction cMRF, and the novel multi‐echo DIP cMRF reconstruction proposed in this work.

Examples of conventional maps and rosette cMRF data reconstructed using direct gridding, iterative low‐rank reconstruction, and the DIP reconstruction in one subject are shown in Figure 7. For each image, the mean and voxel‐level standard deviation of values within an ROI covering the myocardium are reported. In general, the iterative low‐rank method leads to a reduction in the noise (as measured by voxel‐level standard deviation) compared to the direct reconstruction, although elevated T_1_ and T_2_ values may arise due to interactions with the blood pool; the multi‐echo DIP reconstruction is able to further reduce the standard deviation of the measurements while preserving the boundaries between blood and myocardium.

**FIGURE 8:**
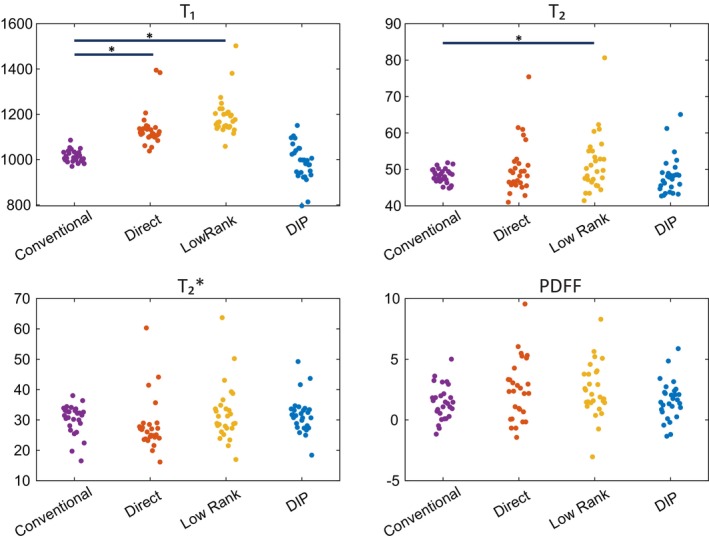
Scatter plots of tissue property values of the healthy subjects within a myocardial ROI for each reconstruction type. Populations with mean values that are significantly different from the conventional scans with *p* < 0.05 are marked with (*).

Figure 8 shows scatter plots of myocardial values for the study population, comparing the measurements made using conventional scans to rosette cMRF with each reconstruction. A table of mean values, and voxel‐level standard deviation of tissue property values is provided in Table S2. For each of tissue property measured, the difference between DIP‐reconstructed rosette cMRF and conventional methods is not statistically significant. However, the direct reconstruction showed a statistically significant difference compared to the conventional methods in T_1_ (mean: 122 ms, CI: 92–153 ms, Cohen's D: 2.05) and the iterative low‐rank reconstruction showed statistically significant differences in T_1_ (mean: 176 ms, CI: 144–209 ms, Cohen's D: 2.8) and T_2_ (mean: 3.5 ms, CI: 0.7–6.2 ms, Cohen's D: 0.59).

**FIGURE 9:**
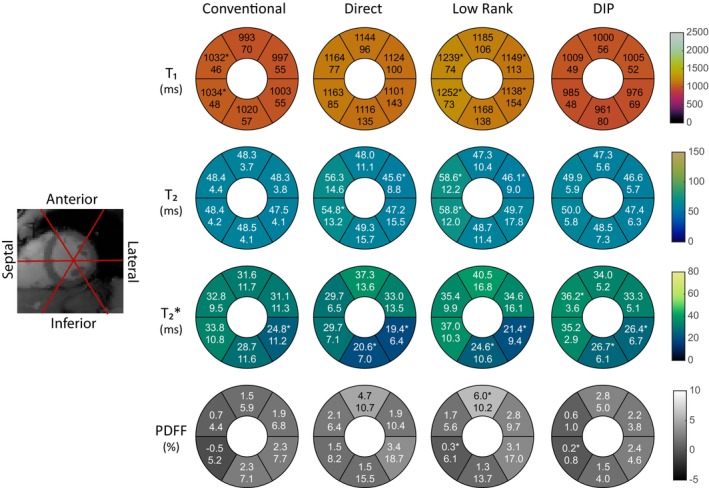
Bullseye plots with tissue property values for each mid‐ventricular AHA segment of the left ventricle, for each reconstruction. Mean values (upper number) and mean voxel‐level SDs (lower number) are reported for each segment; segment measurements that differ significantly from the whole‐myocardium measurement are marked with an asterisk. With the iterative low‐rank reconstruction, septal T_1_ and T_2_ values tend to be higher, potentially due to motion artifacts and influence of the blood pool. A decrease in $T_{2}^{*}$ is observed in the inferior and inferolateral segments across all measurement methods due to susceptibility issues in the region.

Figure 9 shows mean values and mean voxel‐level standard deviations for each myocardial segment and reconstruction type over the study population, compared to conventional measurements. In T_1_, the conventional and iterative low‐rank methods show a significant difference in the septal regions, in both cases higher than the global measurements. The low‐rank method shows a significant decrease in the lateral regions. In T_2_, the direct and low‐rank methods show an increase in at least one of the septal regions; the low‐rank method shows a decrease in the anterolateral segment. In $T_{2}^{*}$, each reconstruction method shows a decrease in the inferior and/or inferolateral segment, and the DIP reconstruction shows an increase in the anteroseptal region. In PDFF, the iterative low‐rank and DIP reconstructions show a decrease in the inferoseptal region, and the low‐rank method shows an increase in the anterior region.

Figure S4 shows a set of Bland–Altman analyses comparing the values measured in healthy subjects with direct, iterative low‐rank, and DIP rosette cMRF to reference MOLLI, T_2_‐prep bSSFP, and GRE scans. The range of the 95% limits of agreement was smallest for the direct reconstruction for T_1_ and smallest for the DIP reconstruction for T_2_, $T_{2}^{*}$, and PDFF. For every tissue property, DIP showed the smallest bias.

**FIGURE 10:**
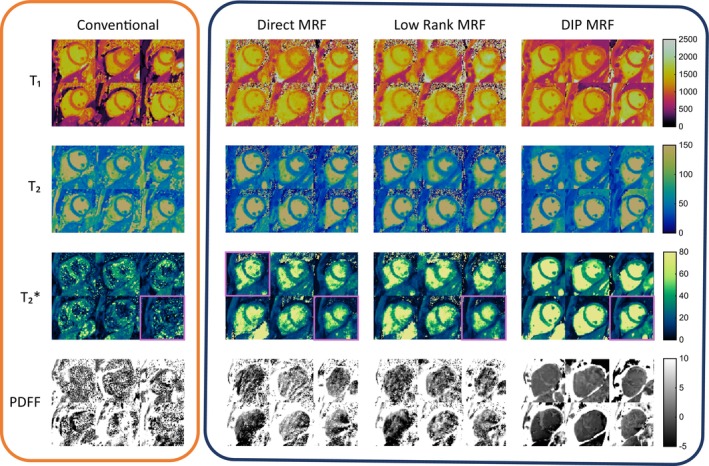
Maps showing T_1_, T_2_, $T_{2}^{*}$, and PDFF from six subjects selected from the 28 enrolled in this study. Subjects with susceptibility artifacts in $T_{2}^{*}$ are highlighted in pink.

Figure 10 depicts water T_1_, water T_2_, water $T_{2}^{*}$, and fat fraction maps collected in 6 of the 28 healthy subjects, which demonstrate the performance of the rosette cMRF approach in a variety of cases. A susceptibility artifact appears on four of the $T_{2}^{*}$ maps reconstructed using the DIP approach, although this artifact also appears on $T_{2}^{*}$ maps from the direct and iterative low‐rank reconstructions, as well as in two of the conventional scans.

## 4 Discussion

In this work, a cMRF approach to simultaneously measure T_1_, T_2_, $T_{2}^{*}$, and PDFF using a deep image prior reconstruction adapted for multi‐echo cMRF data is introduced. The proposed acquisition and DIP reconstruction are compared to both conventional myocardial tissue property mapping methods and direct and iterative locally low‐rank cMRF reconstructions. In standard phantoms and human subjects, rosette cMRF with DIP reconstruction shows good agreement with conventional mapping techniques. Compared to other cMRF reconstruction techniques in healthy subjects, the DIP reconstruction shows lower voxel‐level standard deviations for all measured tissue properties.

Previous works on multi‐echo cardiac MRF for T_1_, T_2_, $T_{2}^{*}$, and PDFF mapping acknowledge drawbacks such as limited resolution, long breathhold requirements, and errors introduced from through‐plane motion [17]. In this work, these issues are addressed using a rosette trajectory sequence with a higher spatial resolution, shorter breathhold, and a shorter cardiac acquisition window (which reduces the need for motion correction). Due to the reduced number of TRs used in this sequence compared with other cMRF implementations, maps generated via direct matching and iterative low‐rank reconstruction show higher voxel‐level standard deviations in measurements compared to conventional maps. The DIP reconstruction proposed to combat these challenges demonstrates agreement with conventional mapping approaches, and superior performance compared to direct and iterative low‐rank methods in terms of the voxel‐level standard deviation of the measurements. These effects are highlighted in Figure 7, where a comparison between each reconstruction method is shown for a representative subject. In the direct reconstruction, a substantial amount of noise is present, causing an increase in the voxel‐level standard deviation of tissue property measurements in the septal region. The iterative low‐rank reconstruction yields a reduced voxel standard deviation in the measurements but increases the mean tissue property values for the septal region. The deep image prior reconstruction is able to reduce the voxel‐level variance of the tissue property measurements in the septal myocardium, while maintaining a similar mean value compared to the conventional scans.

### 4.1 Phantoms

In the MRXCAT phantom, measurements made with rosette cMRF agree with the ground‐truth values used in simulation. In the NIST phantom, there is excellent agreement between measurements made using rosette cMRF with the DIP reconstruction and reference measurements for T_1_ and T_2_ properties. The T_2_ values measured using rosette cMRF in the NIST phantom are in better agreement with the values measured using the spin‐echo sequence compared to previous fat/water separated cMRF studies at 1.5 T [14, 36], where values measured using cMRF were lower compared to those measured using spin‐echo. This difference may be attributed to the ability to correct for $T_{2}^{*}$ effects, which could lead to inaccuracies in T_2_ in other cMRF implementations. The $T_{2}^{*}$ values measured using cMRF and gold‐standard GRE also show excellent agreement in the NIST phantom for values up to 40 ms^1^. The fat fraction values measured using cMRF and GRE also show excellent agreement over the full range of fraction values [8].

### 4.2 Healthy Subjects

Water‐ and fat‐separated T_1_, T_2_, and $T_{2}^{*}$ maps, and PDFF maps were successfully collected in all 28 healthy subjects using rosette cMRF. Both *t*‐tests and Bland–Altman analysis show that the values measured with rosette cMRF are in good agreement with the clinical reference methods. For the proposed DIP reconstruction, there are no significant differences in the mean tissue property value measured in healthy subjects compared to current clinical mapping sequences. Significant differences are only observed in conventional cMRF reconstruction techniques. For T_1_, MOLLI measurements are highly dependent on the imaging parameters and reconstruction techniques used [1, 37]; it is observed that the direct reconstruction results in estimates consistent with previously reported cMRF values [17], and it is known that the DIP yields T_1_ estimates slightly lower compared to other cMRF reconstructions [19]. While spiral cMRF T_2_ values are generally underestimated compared to T_2_‐prep bSSFP [12], rosette cMRF T_2_ values show better agreement, potentially due to correction of $T_{2}^{*}$ effects. *T*‐tests and Bland–Altman analysis of $T_{2}^{*}$ measurements indicate good agreement between $T_{2}^{*}$ measurements made using rosette cMRF and gold‐standard GRE sequences. Myocardial fat fraction measurement in the healthy subjects, where the PDFF is less than 5% [5], can be challenging to measure, as the presence of noise can make it difficult to precisely capture low PDFF values. Overall, this explains the trend seen in the PDFF Bland–Altman plot; measurements with both cMRF and GRE estimate the PDFF to be approximately 5%, but the inter‐subject variance of the cMRF measurement over the population is substantially higher. In general, in vivo measurements of T_1_/T_2_/$T_{2}^{*}/\text{PDFF}$ made with rosette cMRF have a higher inter‐subject variance compared to conventional techniques, indicating that the proposed method is less precise. By simultaneously mapping four properties, the rosette cMRF sequence spends less time encoding each property compared to the conventional sequences, which dedicate an entire acquisition to a single property. It may be expected that this reduction of encoding power may result in a loss of SNR, although this is only observed in T_1_ (cMRF voxel‐level SD 79 vs. 64 ms conventional) and T_2_ (cMRF voxel‐level SD 7.5 vs. 4.4 ms conventional), while the voxel‐level SD is reduced for $T_{2}^{*}$ and PDFF. However, the DIP exhibited smaller Bland–Altman limits of agreement in T_2_, $T_{2}^{*}$, and PDFF compared to the direct and iterative low‐rank reconstructions, indicating that it is more precise compared to other reconstruction methods. Compared to previous publications using multi‐echo cMRF [13, 17], the DIP reconstruction shows lower T_1_ (989 ± 80 vs. 1081 ± 31.8, 1148 ms), higher T_2_ (48.3 ± 5.1 ms vs. 40.5 ± 1.4, 42.8 ms), similar $T_{2}^{*}$ (31.7 ± 5.8 vs. 35.0 ms), and similar PDFF (1.6% ± 1.6% vs. 0.4%, 0.8%).

When divided into the AHA segments, the conventional and iterative low‐rank reconstructions show an increase in T_1_ in the septal regions, potentially due to motion causing the blood pool to enter the septal ROI for a portion of the scan; a similar pattern occurs in the direct and low‐rank reconstructions in T_2_. In these cases, a significantly lower measurement value is observed in the lateral segments as values measured in the septal regions increase the values measured across the whole myocardium. With every method, a decrease in $T_{2}^{*}$ in the inferior and/or inferolateral segments can be seen, potentially due to susceptibility issues in those regions. Finally, the DIP and iterative low‐rank reconstructions show a decrease in inferoseptal PDFF. As in T_1_ and T_2_, this could also potentially be caused by motion.

The $T_{2}^{*}$ maps in five of the 28 subjects collected using rosette cMRF and reconstructed using DIP exhibited a susceptibility artifact causing a signal dropout in the inferior section of the myocardium and the superior section of the liver. This area is a common place for susceptibility artifacts due to the boundary between the lung, liver, heart, and coronary sinus, and has been documented in previous publications [38, 39]. Mitigating this specific artifact is an active research area. Atalay et al. [38] have determined that shimming techniques are unable to resolve this artifact. While reducing the TE may help in $T_{2}^{*}$‐weighted imaging, it is not feasible in $T_{2}^{*}$ mapping, where longer echo times are required to stratify healthy patients from those with mild iron overload. Reduction of slice thickness or re‐orientation of the slice to reduce *χ*‐variation along the slice may be effective in mitigating the artifact but come with additional drawbacks in terms of SNR or non‐conventional slice placement, respectively [38]. In cases where the susceptibility artifact occurred in the DIP reconstruction, it also occurred in either the direct or iterative low‐rank reconstruction. The artifact also appears in five of the reference $T_{2}^{*}$ scans. Compared to previous multi‐echo cMRF studies [17], the higher maximum TE used in this study potentially makes this artifact more visible and frequent.

### 4.3 Limitations

One limitation of this work is that this approach has not yet been tested on a patient population, specifically those with reduced myocardial $T_{2}^{*}$ due to iron overload or increased PDFF due to myocardial fat infiltration. It has been noted that the 300 mm FOV may be too small to accommodate larger patients. The rosette trajectory, like a radial acquisition, tends to be more robust to aliasing artifacts; in addition, some degree of aliasing is expected in MRF acquisitions, and it is expected that the pattern‐matching approach can identify the underlying signal evolutions through these aliasing errors. However, none of the subjects in this study exceeded the 300 mm imaging field, and additional validation of the proposed sequence should be performed on this patient population before clinical deployment. The diastolic acquisition window of 245 ms used in this work may also be too long for patients with rapid heart rates. Reducing the acquisition window may have an impact on map quality, but could still be sufficient to reconstruct maps; additional research is required to determine performance in these cases. In addition, these maps have not been reviewed by cardiologists or radiologists to evaluate their diagnostic quality.

## 5 Conclusions

In this work, a novel DIP method for reconstructing multi‐echo cMRF data is introduced and paired with a single‐breathhold, ECG‐gated rosette cardiac MRF acquisition to simultaneously map T_1_, T_2_, $T_{2}^{*}$, and fat fraction. The proposed method is compared to conventional cardiac mapping scans as well as existing reconstructions in simulations, phantoms, and healthy subjects. The proposed DIP cMRF reconstruction shows good quantitative agreement with conventional methods and offers improved precision over existing cMRF reconstruction techniques.

## Funding

This work was supported by the National Institutes of Health, R01HL153034, R01HL163030, R01HL163991, and Siemens Healthineers.

## Conflicts of Interest

The authors of this work receive research support from Siemens Healthineers.

## Supporting information


**Figure S1:** Diagram of the neural network architecture. A 32‐channel tensor of noise is generated and passed into the network. It is passed through five encoding layers, each consisting of a pair of 128‐channel 3 × 3 2D convolution operations, followed by a 2× downsampling operation. This is then passed through a set of 5 decoding layers, each consisting of a 2× upsampling operation, concatenation with the skipped connection, attention operation (ReLU activation, channel‐wise scaling, and sigmoid activation), a single 128‐channel 3 × 3 2D convolution, and then a channel‐wise scaling operation. After the five decoding layers, the image has returned to the original resolution, and output as the MRF subspace‐echo images used for map reconstruction.
**Figure S2:** The first subspace/first echo image reconstructed using DIP for the first 50 iterations, in 5‐iteration intervals, are shown in the top rows. The first row shows reconstructions that include *B*_0_ corrections in the DIP forward model, while the second row shows reconstructions that omit the *B*_0_ correction step. First, inclusion of the *B*_0_ map enables the DIP network to reconstruct coherent images at a lower iteration number, indicating that it accelerates and stabilizes network training compared to the uncorrected case. Second, this volunteer had a minor susceptibility artifact in the inferior myocardium; without *B*_0_ correction, this artifact is more severe, and causes distortion in the inferolateral region. The plot at the bottom shows RMSE values for each image compared to the reference 100‐iteration reconstruction. The *B*_0_‐corrected reconstruction achieves a given reconstruction quality (measured by RMSE) at a lower iteration number than the reconstruction without *B*_0_ correction.
**Table S1:** Table of imaging parameters for clinical standard mapping methods, used to measure reference values for in vivo scans.
**Figure S3:** Numerical simulations were used to determine the influence of confounding effects of off‐target magnetic properties on the measurements made using the multi‐echo cMRF acquisition coupled with the direct, iterative low‐rank, and DIP reconstructions. A simulated phantom containing 36 vials was generated, with each combination of T_1_ = (300, 800, 1200), T_2_ = (30, 70, 150), $T_{2}^{*}$ = (15, 30), and PDFF = (5%, 15%). The relaxation values of T_1_ = 250, T_2_ = 60, and $T_{2}^{*}$ = 20 ms were used for the fat component. A rosette cMRF acquisition was simulated using this phantom, including off‐resonance and coil sensitivity effects. Additive complex noise with a standard deviation of 2.5% of the maximum signal value was added to the simulated k‐space data. The simulated rosette cMRF data were processed using pattern matching alone, the iterative low‐rank reconstruction, and the DIP processing pipeline. The reconstructed maps are shown on the left, and heat maps depicting the correlation coefficient are shown on the right. For each reconstruction, a statistically significant dependence (marked with an asterisk) is observed between the measurement of each property and the value of the same property used in simulation, as shown along the diagonal of the heat maps. A weak dependence of PDFF on $T_{2}^{*}$ is also observed for each reconstruction, although this dependence is not statistically significant at the *p* < 0.05 level. Inspection of the iterative low‐rank $T_{2}^{*}$ map reveals spatially‐dependent variation, particularly among the $T_{2}^{*}$ = 30 ms group, likely due to residual aliasing artifacts corrupting the reconstruction. This effect is not seen in the direct or DIP reconstructions, indicating that these reconstruction approaches may yield more accurate results.
**Table S2:** Table of the average tissue property value and average voxel‐level standard deviation (SD) within the myocardial ROI for each reconstruction type. While measurements made using cMRF with the DIP reconstruction yield a higher standard deviation across the population compared to the conventional methods, the DIP‐cMRF method is more precise compared to both direct and iterative low‐rank cMRF reconstructions. In addition, the DIP offers a lower average standard deviation in each myocardial measurement compared to the direct and iterative low‐rank reconstructions; while the DIP‐cMRF demonstrates a lower inter‐subject precision compared to the conventional scan for measurements of T_1_ and T_2_, it shows a higher level of inter‐subject precision compared to conventional measurements of $T_{2}^{*}$ and PDFF.
**Figure S4:** Bland–Altman plots comparing T_1_, T_2_, $T_{2}^{*}$, and PDFF values measured using conventional approaches and rosette cMRF reconstructed with direct, iterative low‐rank, and DIP.

## Data Availability

Research data are not shared.
