# Supplementary material for: Rosette Cardiac MR Fingerprinting for Simultaneous T1 , T2 , T2*, and Fat Fraction Mapping Using a Multi‐Echo Deep Image Prior Reconstruction
Source: Magn Reson Med. 2026 Feb 9;95(6):3284–97. doi: 10.1002/mrm.70299 (PMC13049243; doi:10.1002/mrm.70299)
Supplement: Supplementary file 1 — Figure S1: Diagram of the neural network architecture. A 32‐channel tensor of noise is generated and passed into the network. It is passed through five encoding layers, each consisting of a pair of 128‐channel 3 × 3 2D convolution operations, followed by a 2× downsampling operation. This is then passed through a set of 5 decoding layers, each consisting of a 2× upsampling operation, concatenation with the skipped connection, attention operation (ReLU activation, channel‐wise scaling, and sigmoid activation), a single 128‐channel 3 × 3 2D convolution, and then a channel‐wise scaling operation. After the five decoding layers, the image has returned to the original resolution, and output as the MRF subspace‐echo images used for map reconstruction. Figure S2: The first subspace/first echo image reconstructed using DIP for the first 50 iterations, in 5‐iteration intervals, are shown in the top rows. The first row shows reconstructions that include B 0 corrections in the DIP forward model, while the second row shows reconstructions that omit the B 0 correction step. First, inclusion of the B 0 map enables the DIP network to reconstruct coherent images at a lower iteration number, indicating that it accelerates and stabilizes network training compared to the uncorrected case. Second, this volunteer had a minor susceptibility artifact in the inferior myocardium; without B 0 correction, this artifact is more severe, and causes distortion in the inferolateral region. The plot at the bottom shows RMSE values for each image compared to the reference 100‐iteration reconstruction. The B 0‐corrected reconstruction achieves a given reconstruction quality (measured by RMSE) at a lower iteration number than the reconstruction without B 0 correction. Table S1: Table of imaging parameters for clinical standard mapping methods, used to measure reference values for in vivo scans. Figure S3: Numerical simulations were used to determine the influence of confounding effects of off‐target ma [file MRM-95-3284-s001.docx]

# Supporting Material


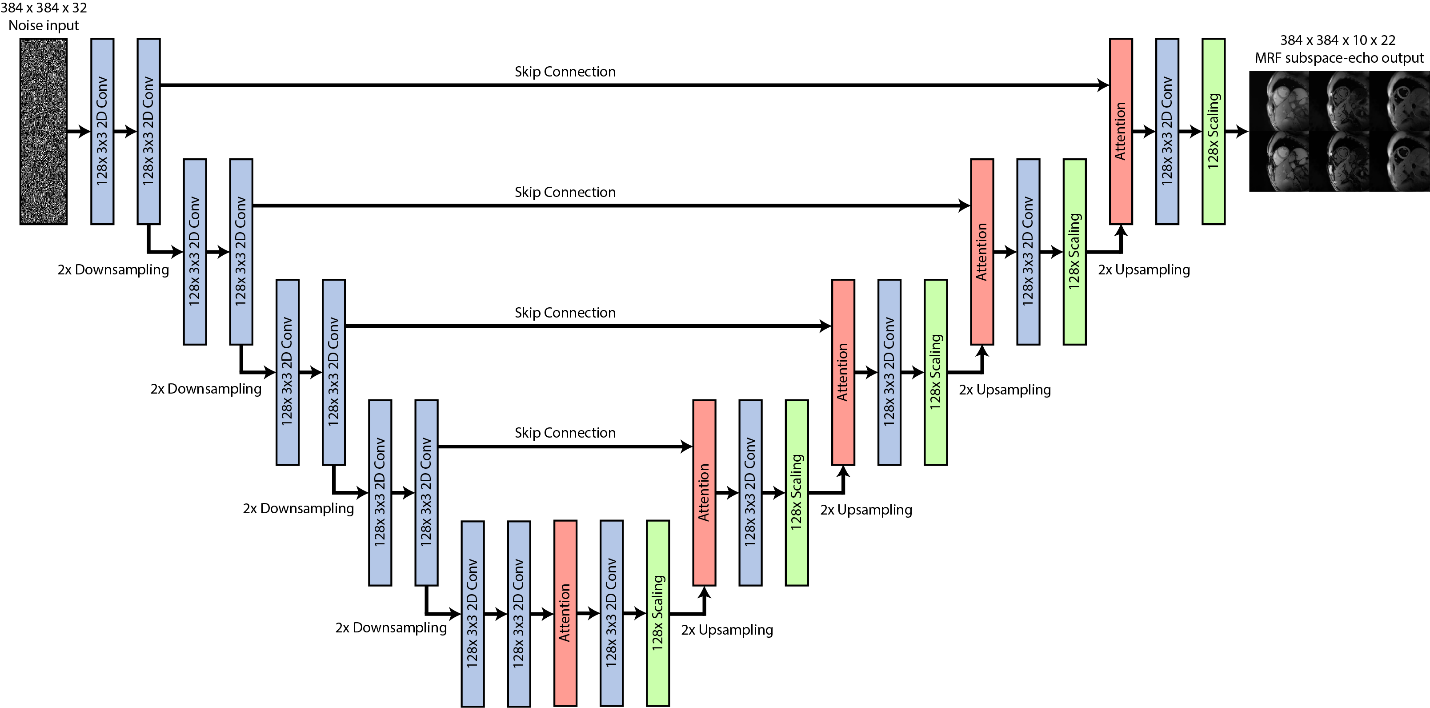


**Figure S1.** Diagram of the neural network architecture. A 32-channel tensor of noise is generated and passed into the network. It is passed through five encoding layers, each consisting of a pair of 128-channel 3x3 2D convolution operations, followed by a 2x downsampling operation. This is then passed through a set of 5 decoding layers, each consisting of a 2x upsampling operation, concatenation with the skipped connection, attention operation (ReLU activation, channel-wise scaling, and sigmoid activation), a single 128-channel 3x3 2D convolution, and then a channel-wise scaling operation. After the five decoding layers, the image has returned to the original resolution, and output as the MRF subspace-echo images used for map reconstruction.

**
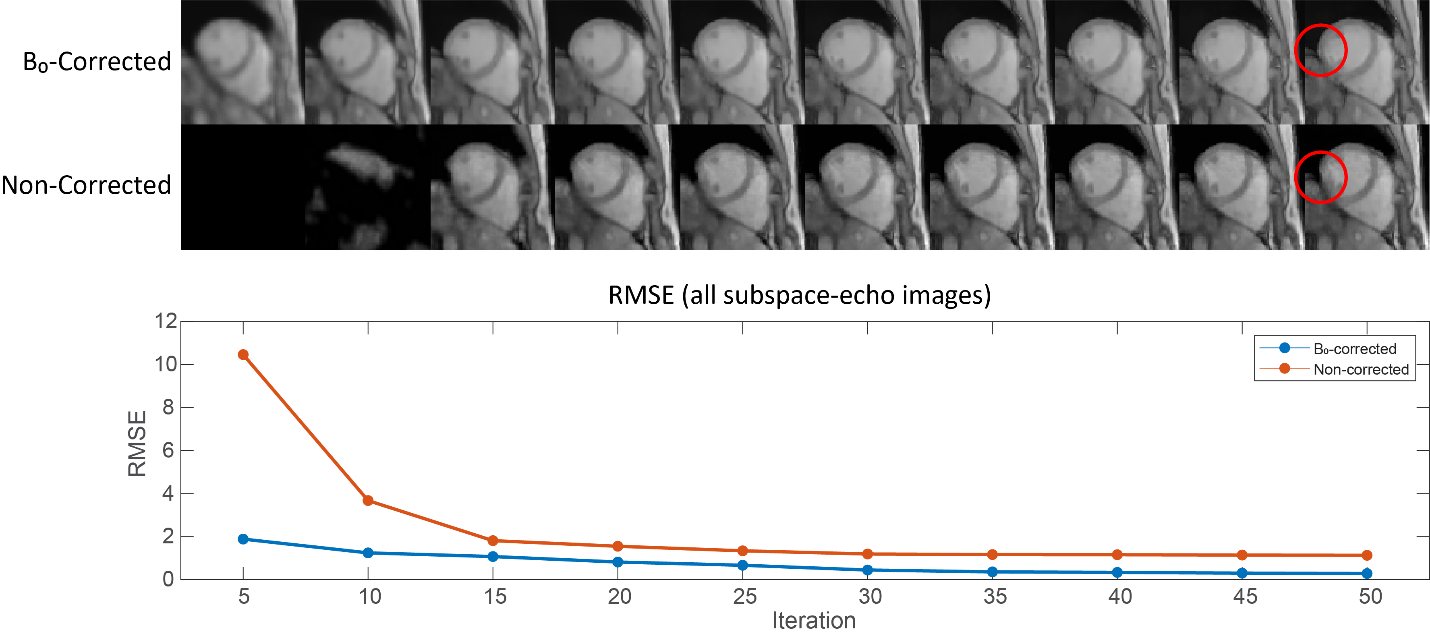
**

**Figure S2.** The first subspace/first echo image reconstructed using DIP for the first 50 iterations, in 5-iteration intervals, are shown in the top rows. The first row shows reconstructions that include B_0_ corrections in the DIP forward model, while the second row shows reconstructions that omit the B_0_ correction step. First, inclusion of the B_0_ map enables the DIP network to reconstruct coherent images at a lower iteration number, indicating that it accelerates and stabilizes network training compared to the uncorrected case. Second, this volunteer had a minor susceptibility artifact in the inferior myocardium; without B0 correction, this artifact is more severe, and causes distortion in the inferolateral region. The plot at the bottom shows RMSE values for each image compared to the reference 100-iteration reconstruction. The B_0_-corrected reconstruction achieves a given reconstruction quality (measured by RMSE) at a lower iteration number than the reconstruction without B_0_ correction.

|  | MOLLI T1 | T2-prep bSSFP | GRE T2*/PDFF |
| --- | --- | --- | --- |
| FOV (Read) | 300 | 300 | 300 |
| FOV (Phase) | 240 | 240 | 300 |
| Slice Thickness | 8 | 8 | 8 |
| TR (echo spacing) | 2.61 | 2.67 | 18.65 |
| TE | 1.08 | 1.14 | 2.56/4.58/6.60/8.62/10.64/12.66/14.68/16.70 |
| TI/T2TE | 181 | 0/25/55 |  |
| FA | 35 | 70 | 20 |
| Resolution (Read) | 192 | 192 | 192 |
| Resolution (Phase) | 192 | 192 | 192 |
| Reconstruction | GRAPPA | GRAPPA | GRAPPA |
| R | 2 | 2 | 2 |
| Reference | GRE/Separate | GRE/Separate | Integrated |
| Reference Lines | 36 | 36 | 24 |
| Partial Fourier | 7/8 | 6/8 | Off |
| BW | 1085 Hz/Px | 1184 Hz/Px | 814 Hz/Px |

**Table S1.** Table of imaging parameters for clinical standard mapping methods, used to measure reference values for in vivo scans.


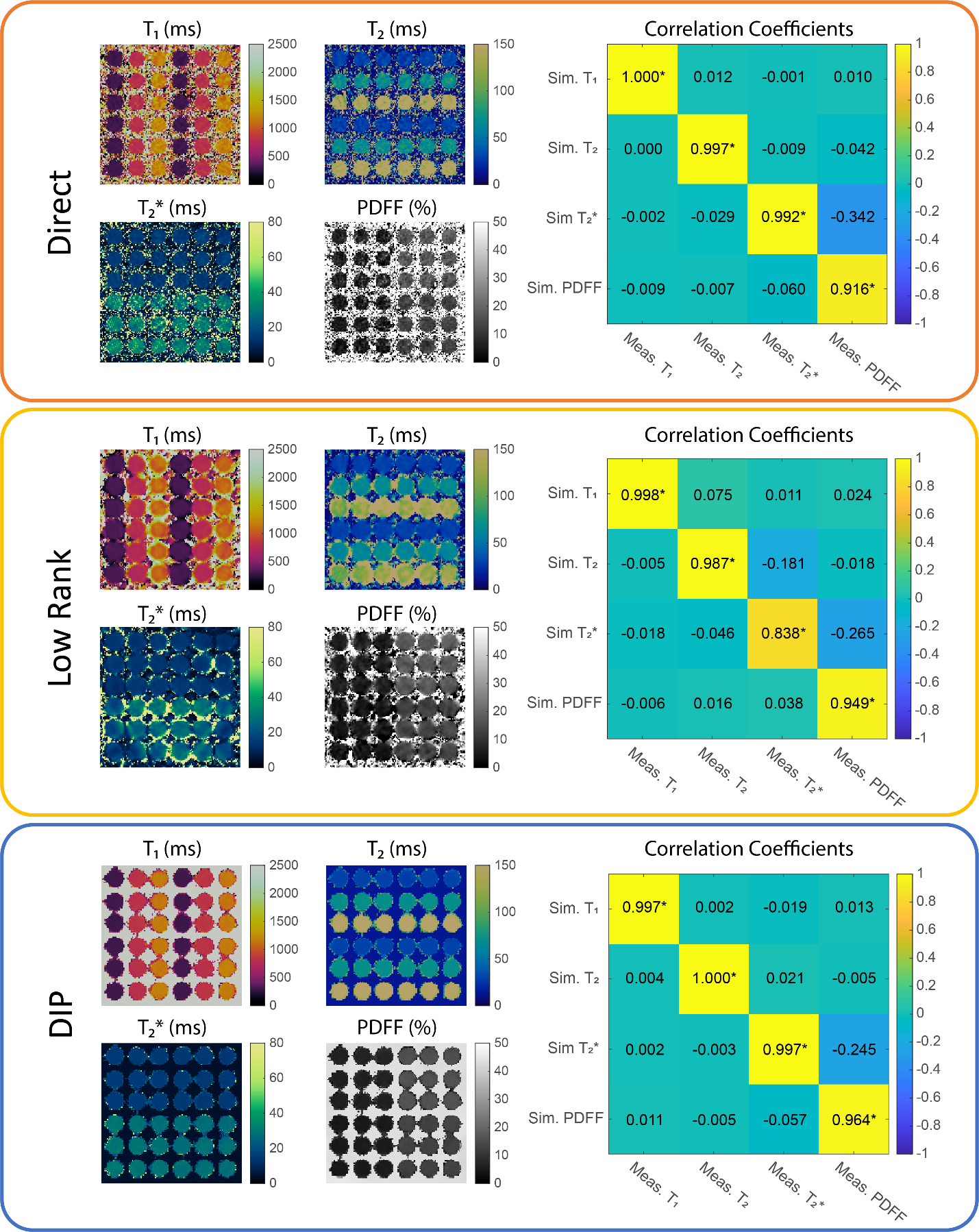


**Figure S3.** Numerical simulations were used to determine the influence of confounding effects of off-target magnetic properties on the measurements made using the multi-echo cMRF acquisition coupled with the direct, iterative low-rank, and DIP reconstructions. A simulated phantom containing 36 vials was generated, with each combination of T_1_ = [300 800 1200], T_2_ = [30 70 150], T_2_* = [15 30], and PDFF = [5% 15%]. The relaxation values of T_1_ = 250, T_2_ = 60, and T_2_* = 20 ms were used for the fat component. A rosette cMRF acquisition was simulated using this phantom, including off-resonance and coil sensitivity effects. Additive complex noise with a standard deviation of 2.5% of the maximum signal value was added to the simulated k-space data. The simulated rosette cMRF data were processed using pattern matching alone, the iterative low-rank reconstruction, and the DIP processing pipeline. The reconstructed maps are shown on the left, and heat maps depicting the correlation coefficient are shown on the right. For each reconstruction, a statistically significant dependence (marked with an asterisk) is observed between the measurement of each property and the value of the same property used in simulation, as shown along the diagonal of the heat maps. A weak dependence of PDFF on T_2_* is also observed for each reconstruction, although this dependence is not statistically significant at the *p*<0.05 level. Inspection of the iterative low-rank T_2_* map reveals spatially-dependent variation, particularly among the T_2_* = 30 ms group, likely due to residual aliasing artifacts corrupting the reconstruction. This effect is not seen in the direct or DIP reconstructions, indicating that these reconstruction approaches may yield more accurate results.

|  | T1 mean | T1 SD | T2 mean | T2 SD | T2* mean | T2* SD | PDFF mean | PDFF SD |
| --- | --- | --- | --- | --- | --- | --- | --- | --- |
| Conventional | 1014 ± 25 ms | 64 ± 16 ms | 48.3 ± 1.9 ms | 4.4 ± 0.9 ms | 30.3 ± 4.7 ms | 13.2 ± 3.4 ms | 1.4 ± 1.4% | 7.2 ± 3.3% |
| Direct cMRF | 1137 ± 78 ms* | 122 ± 43 ms | 50.3 ± 7.1 ms | 17.4 ± 13.3 ms | 28.0 ± 8.3 ms | 13.7 ± 12.0 ms | 2.5 ± 2.5% | 13.9 ± 9.2% |
| LLR cMRF | 1191 ± 83 ms* | 137 ± 51 ms | 51.7 ± 7.8 ms* | 16.1 ± 11.4 ms | 32.0 ± 9.0 ms | 17.1 ± 11.9 ms | 2.5 ± 2.2% | 12.3 ± 10.8% |
| DIP cMRF | 989 ± 80 ms | 79 ± 26 ms | 48.3 ± 5.1 ms | 7.5 ± 2.9 ms | 31.7 ± 5.8 ms | 7.8 ± 2.2 ms | 1.6 ± 1.6% | 4.5 ± 2.4% |

**Table S2.** Table of the average tissue property value and average voxel-level standard deviation (SD) within the myocardial ROI for each reconstruction type. While measurements made using cMRF with the DIP reconstruction yield a higher standard deviation across the population compared to the conventional methods, the DIP-cMRF method is more precise compared to both direct and iterative low-rank cMRF reconstructions. In addition, the DIP offers a lower average standard deviation in each myocardial measurement compared to the direct and iterative low-rank reconstructions; while the DIP-cMRF demonstrates a lower inter-subject precision compared to the conventional scan for measurements of T_1_ and T_2_, it shows a higher level of inter-subject precision compared to conventional measurements of T_2_* and PDFF.


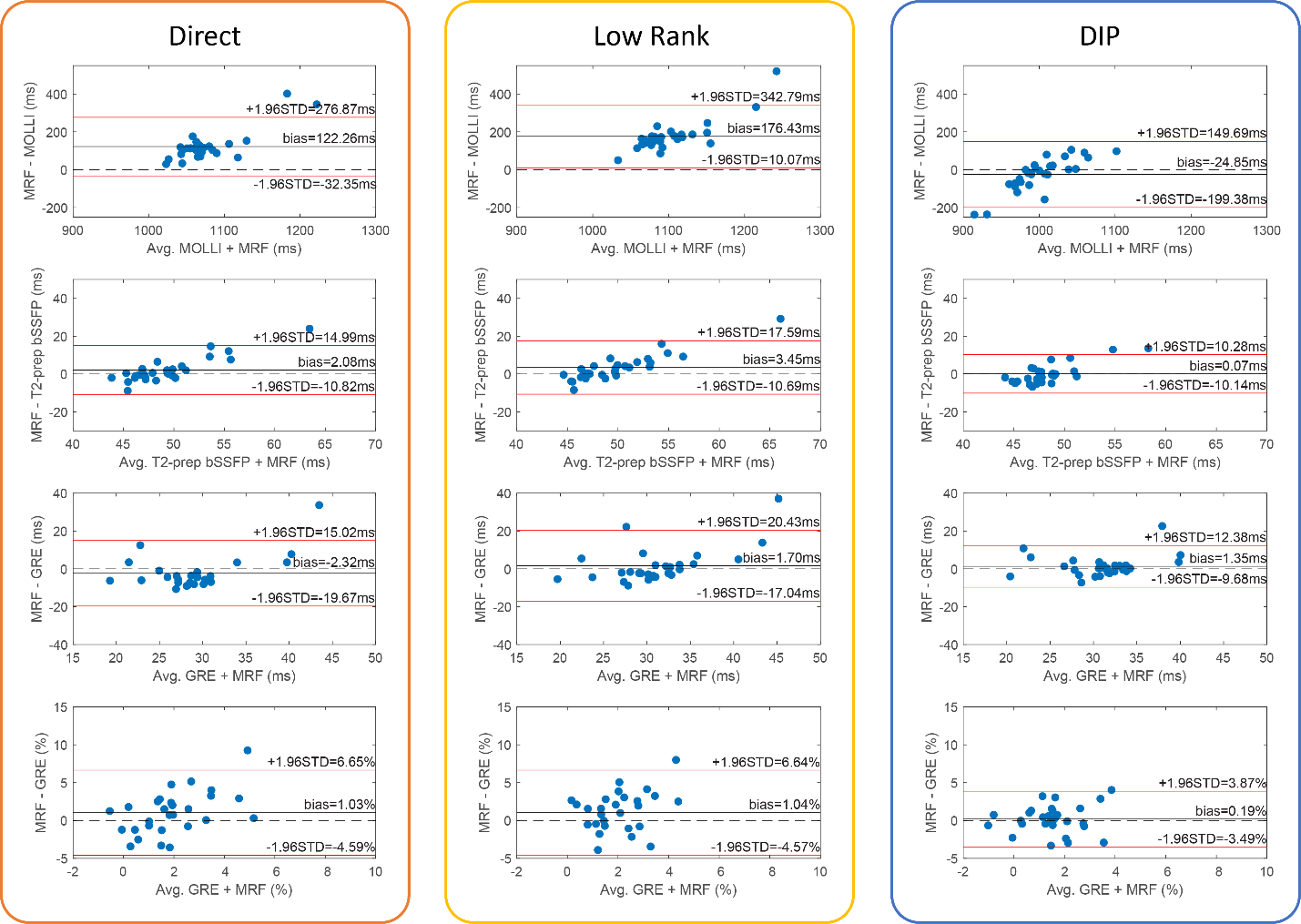


**Figure S4.** Bland-Altman plots comparing T_1_, T_2_, T_2_*, and PDFF values measured using conventional approaches and rosette cMRF reconstructed with direct, iterative low-rank, and DIP.
